# Sound reception and hearing capabilities in the Little Penguin (*Eudyptula minor*): first predicted in-air and underwater audiograms

**DOI:** 10.1098/rsos.240593

**Published:** 2024-08-28

**Authors:** Chong Wei, Christine Erbe

**Affiliations:** ^1^ Centre for Marine Science & Technology, Curtin University, GPO Box U1987, Perth, Western Australia 6845, Australia

**Keywords:** hearing, sound reception, little penguin, finite element, microCT scan

## Abstract

Despite increasing concern about the effects of anthropogenic noise on marine fauna, relevant research is limited, particularly in those inaccessible species, such as the Little Penguin (*Eudyptula minor*). In this study, we collected freshly deceased Little Penguins for dissection and micro-computed tomography (microCT) scans. The head structures, including the ear apparatus, were reconstructed based on high-resolution imaging data for the species. Moreover, three-dimensional finite-element models were built based on microCT data to simulate the sound reception processes and ear responses to the incident planar waves at the selected frequencies. The received sound pressure fields and motion (i.e. displacement and velocity) of the internal ear-related structures were modelled. The synergistic response of ear components to incident aerial and underwater sounds was computed to predict the hearing capabilities of the Little Penguins across a broad frequency range (100 Hz–10 kHz), both in air and under water. Our predicted data showed good agreement with other diving birds in both the form and range of auditory sensitivity. This study demonstrates a promising method to study hearing in other inaccessible animals. The outputs from this study can inform noise impact mitigation and conservation management.

## 1. Introduction

Research regarding the effects of anthropogenic noise on seabirds is limited, particularly in penguins. The Australian Little Penguin (*Eudyptula minor*), also called the Fairy Penguin, Little Blue Penguin or Blue Penguin, ranges from southern Western Australia, along Australia’s south coast, to the New South Wales/Queensland border in the east, as well as along the Tasmanian coast and most of New Zealand. Their numbers are declining at many of the colonies. Food availability [1] and loss of foraging habitat are two main concerns regarding their decline. Sound is an important feature of habitat. Previous studies found that areas exposed to high levels of noise may represent habitat loss for some species, causing a decline in species’ presence and density [2]. For example, birds avoid areas with high noise amplitudes [3]. Research also suggested that noise reduces the quality of seabird habitats, leading to progressively acoustic habitat degradation for seabirds [4]. This is because noise interferes with the ability to hear predators, prey and other important sound sources in the environment [5]. Many seabirds find food resources through listening [6], and so noise could disrupt feeding and cause changes in foraging behaviour. Penguins are spheniscids (non-alcid diving birds). Foraging dives of some penguins can be as deep and long as in some species of seals at mesopelagic depths [7]. A recent study [8] showed that Gentoo Penguins (*Pygoscelis papua*) can detect and react to underwater (UW) sound, suggesting the penguins are likely to make use of sound for orientation and prey detection during dives, and they may be very sensitive to anthropogenic noise (e.g. the noise from ships and near-shore construction). The Little Penguin thus is of concern because its critical habitats often overlap with ocean areas used by humans for transport, as well as industrial and recreational purposes. However, to date, the lack of information about Little Penguins sound reception mechanisms and their hearing sensitivity to various acoustic frequencies impedes a thorough assessment of the impacts of anthropogenic noise on these animals. Over the years, only one penguin species, the Black-footed Penguin (*Spheniscus demersus*), also called African Penguin, has been studied with respect to aerial auditory capabilities by using cochlear potential methods (not auditory thresholds) [9].

The conventional methods for estimating an audiogram (i.e. a curve of hearing sensitivity with frequency) can be classified into three categories: (i) predict an audiogram based on the characteristics of the vocalizations (i.e. what animals can hear matches what they can generate) [10]; (ii) predict an audiogram based on ear anatomy and compare with the functional morphology of ears in well-known closely related species [11–13]; and (iii) measure an audiogram through practical methods, such as behavioural response experiments and electrophysiological testing (e.g. auditory brainstem response; ABR) [14]. Unfortunately, these methods are generally not reliable or practical to extract audiograms from Little Penguins, because this species is more elusive, not easily trainable and inaccessible for live testing in a well-designed, controlled laboratory setting.

To overcome the challenges, here we developed sound reception finite-element (FE) models based on high-resolution computed tomography (CT) imaging. To the best of our knowledge, the imaging-based FE modelling and optical coherence tomography (recently introduced as a vibrometry system in auditory research [15] are the two techniques currently available to study the motion patterns (i.e. vibration, displacement, velocity, etc.) of the auditory organs from induced sounds in the small-sized animals. Previous ear morphology studies showed that penguins do not have external ear flaps [16]. Only a pair of ear openings (round/oval holes) are located behind the eyes and covered by feathers. The received sounds are transmitted through the tympanic membrane to the middle ear, and then transferred by the columella (ear ossicle) to the oval window of the inner ear [17]. The inner ear converts the sounds to electronic signals for the nerve cells [16]. The imaging-based FE modelling techniques allow us to further study this sound reception process and to predict audiograms in Little Penguins. The techniques have been used to study the middle ears of multiple species of terrestrial mammals [18–25]. They have also been used to study sound reception and noise impact on multiple marine species, such as fishes [26], baleen whales [24,25,27], and toothed whales [28–30]. The techniques have the advantage of studying the complex acoustic processes when sounds interact with animal biological structures, becoming a useful tool when traditional experimental methods are not possible. Based on the audiograms (in-air (IA) and UW), we may assess potential acoustic impacts on Little Penguins and make relevant mitigation plans, which are critical for conservation efforts.

## 2. Material and methods

### 2.1. Specimen collection and dissection

**Figure 1.**
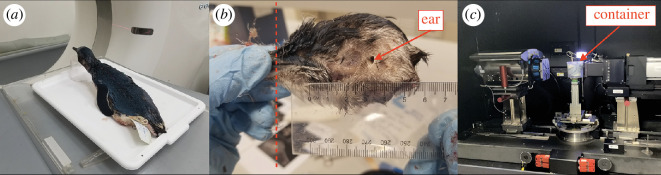
Medical CT and microCT scan. (*a*) Medical CT scanning of a penguin’s head. (*b*) Dissection on the penguin’s head. The red dashed line shows the position where the beak was removed. (*c*) MicroCT scanning of the penguin’s head. The red arrow shows where the plastic container was firmly fixed.

Three full bodies of fresh dead Little Penguins were available from Garden Island on 6 June 2019, 24 September 2019 and 21 February 2023, respectively. All the heads of the specimens were intact. We first performed a medical CT scan on each penguin (figure 1*a*). The specimens were three-dimensionally scanned by X-ray CT using a Siemens SOMATOM definition AS medical scanner installed at the Commonwealth Scientific and Industrial Research Organisation (CSIRO, Curtin Bentley) allowing rapid three-dimensional scanning that produces CT transversal images of 128 × 245 pixels. The scan was conducted using 0.6 mm spiral acquisition at 100 kV and 500 mA and a slice increment of 0.1 mm. All CT data were then imported into the free software Horos™ (Horos Project, Geneva, Switzerland) for CT data analysis and geometrical model reconstruction.

The audiogram is shaped by the external, middle and inner ear connected in series, with the external and middle ear acting as the main contributors to low and mid frequencies for the audiogram [25,31]. Therefore, constructing an accurate ear model with the ear canal, middle ear and inner ear is critical for predicting the audiograms. In the beginning, although we examined multiple CT data based on different individuals/scan settings/reconstruction methods, the columella, tympanic membrane and part of the inner ear (semicircular canals) were too small to be reconstructed in detail owing to the relatively low resolution of the CT data. Therefore, we decided to perform a microCT scan with much higher resolution using one of the penguin’s heads. Owing to the size limitation of the scanner (approx. 40 mm × 40 mm × 40 mm), the beak of the penguin had to be removed from the head without breaking other structures. Therefore, prior to the microCT scan, we conducted a dissection to carefully remove the beak (along the red dashed line in figure 1*b*) and fixed it firmly in a plastic container for microCT scanning, as shown in figure 1*b,c*.

### 2.2. Micro-computed tomography scan and data analysis

MicroCT scanning was performed at the Australian Resources Research Centre (ARRC, Kensington, Western Australia) using an Xradia VersaXRM-500 microCT scanner. The Xradia VersaXRM-500 provides high-resolution three-dimensional X-ray imaging with submicron resolution. It facilitates non-destructive observation of the internal structure of objects, such as the columella. For microCT scanning, the isotropic resolution was set as voxel size 29 µm and the power setting was 80 kV × 87 µA. The slice increment and slice thickness were both 0.05 mm. Cone-beam reconstruction was performed on the object and the data were saved on a hard drive as a matrix of 1000 × 1024 pixels per image in a TIFF format. The images were converted to DICOM format later and imported into Horos™ for data analysis and three-dimensional geometrical model reconstruction.

**Figure 2.**
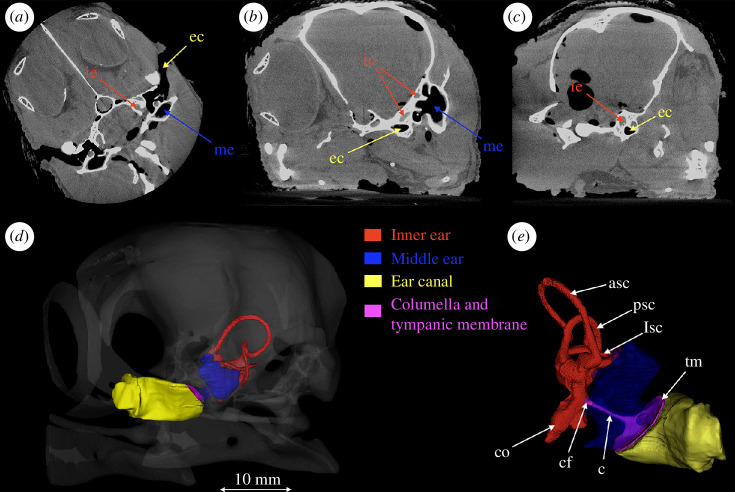
MicroCT scan data and the detailed three-dimensional reconstruction of the left ear. (*a*) Coronal plane, (*b*) nonorthogonal sagittal plane and (*c*) sagittal plane of the head. The grey level represents the different Hounsfield Unit (HU) values. (*d* and *e*) Three-dimensional reconstruction of the skull and ear structures. The ear structure is located in the lower back of the skull. The red region represents the inner ear, the blue region the middle ear, the yellow region the ear canal and the purple region the columella and tympanic membrane [29,30]. The tympanic membrane conveys sound from the ear canal to the middle ear. The bony columella (the purple region in figure 2*e*), the only auditory ossicle of the bird, transfers the vibration through the columella’s footplate to the oval window of the inner ears. Abbreviations: ie, inner ear; me, middle ear; ec, ear canal; co, cochlea; cf, columella’s footplate; c, columella; tm, tympanic membrane; asc, anterior semicircular canal; psc, posterior semicircular canal; lsc, lateral semicircular canal.

The microCT data of the penguin’s head included 1011 slices. The size of the ear structures of the Little Penguin is very small: the length of the inner ear is approximately 14 mm and the length of the columella is approximately 5 mm. To assure the accuracy of the model reconstruction, we had to manually perform the segmentation slice-by-slice based on the penguin anatomy to construct the ear structures. The detailed ear structure three-dimensional reconstruction is shown in figure 2*e*. Figure 2 displays the analysis of the microCT data and three-dimensional reconstruction of the head including the ear structures. All the head structures were exported as a stereolithography (STL) file after multiple steps of optimization and post-processing (i.e., smoothing, removing overlapping and self-intersections, etc.) of the raw data using the software Horos™ and COMSOL Multiphysics (Stockholm, Sweden).

### 2.3. Finite-element sound reception model construction

**Figure 3.**
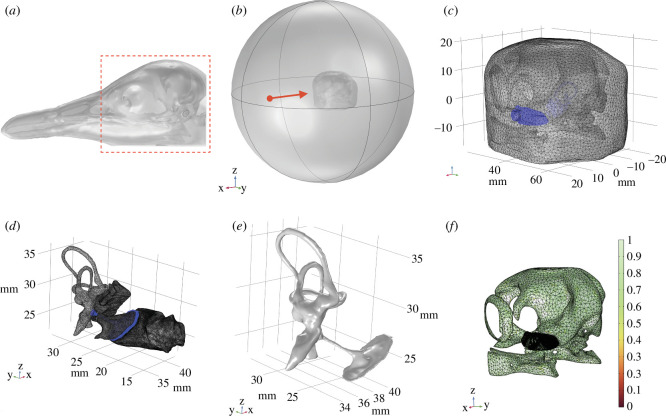
FE model set-ups and meshing. (*a*) The CT scan of the full head. The red frame shows the part that was cut for microCT scanning. (*b*) The set-up of the model based on microCT data. The red points denote the acoustic stimulus and the red arrows show the directions of the incoming sound (parallel to the *x-*axis). (*c*) The meshing of the head for the FE models, the blue region denotes the ear structures. (*d*) The meshing of the ear structures, including the inner ear, middle ear and the ear canal. (*e*) The geometries of the inner ear, columella and tympanic membrane. (*f*) The mesh quality of the FE model (the soft tissues were not shown in this figure for display purposes). Note the different scales on the skull and ear canal. The colour scale shows the quality of the mesh elements, from red (poor) to green (good). The mesh element quality, which measures the regularity of the mesh elements’ shapes, is a dimensionless quantity between 0 and 1, where 1 represents a perfectly regular element, in the chosen quality measure and 0 represents a degenerated element (an element is collapsed by one or more edges).

The STL files were imported into the COMSOL Multiphysics modelling software (Stockholm, Sweden) for finite-element analysis (FEA) and corresponding data analysis. In this research, we studied both IA and UW hearing. Thus, we built two FE sound reception models based on microCT data (IA model and UW model). The set-ups of the models are shown in figure 3.

Two FE models simulated the sound reception process from an outside medium (air or seawater) to the inner ear of a Little Penguin. An air sphere and water sphere were set outside of the head for the IA and UW models, respectively, simulating a Little Penguin receiving sound through air and seawater (figure 3*b*). The diameter of the sphere was set as 200 mm. A low-reflecting boundary condition [32,33] was applied to both spheres, simulating the sound transmitting in free space with minimum boundary reflections. Considering the FE models do not contain the beak, to avoid any impact of missing beak, an incident acoustic wave was set in front of the left ear hole (60 mm from the ear hole on the left side of the head) with a given pressure amplitude for all models. The magnitude of the input pressure was arbitrary since the model was linear. The stimulus was directed towards the left side of the penguin’s head at the selected frequencies, as the red arrow shown in figure 3*b*. The frequency was swept from 100 Hz to 10 kHz in steps of 200 Hz for both the IA and the UW models. The sound wave travelled through the air or seawater surrounding the head of the penguin and then interacted with the complex head structures to generate traction loads on the surface of the ears.

COMSOL’s free mesher was used to generate a free tetrahedral mesh to map the entire model. The free mesh has a built-in detection for small features and narrow regions in a geometry. It also has an automatic element size adjustment for small features, narrow regions and curved boundaries, such as the ear apparatus (figure 3*c,d*). It is widely accepted that the element size in element-based acoustic computations should be related to the wavelength. Often, the element size is measured in a certain (fixed) number of elements per wavelength. For the three-dimensional acoustic modelling, the rule of thumb for meshing wave problems is to apply at least 5−6 second-order mesh elements per local wavelength in order to resolve waves, including resolving elastic waves in the solid [34,35]. In this study, the maximum mesh size was set to at least one-sixth of the wavelength at each frequency for each material (i.e. air, seawater, soft tissue, skull, etc.). To gain confidence in the accuracy of the model, a mesh refinement analysis/mesh convergence study was conducted by resolving the model on progressively finer meshes to find the optimal element size for the model (details about mesh refinement are provided in the electronic supplementary material, file S1). Based on the mesh refinement results, the size of the element for air was set between 1 and 5 mm, the size of the element for seawater was set between 1 and 15 mm, the size of the element for the soft tissues was set between 1 and 15 mm, the size of the element for the bony structures was set between 5 and 30 mm and the size of the element for the ear structures (i.e. ear canal, inner ear, columella, etc.) was set between 1 and 5 mm. The meshing is shown in figure 3*b,c*. For the IA model, the geometry translated to a mesh consisting of 1.79 million tetrahedral elements, resulting in the number of degrees of freedom solved of 2.94 million. For the UW model, the geometry translated to a mesh consisting of 1.37 million tetrahedral elements, resulting in the number of degrees of freedom solved of 2.38 million. The mesh quality is shown in figure 3*f*.

The finite element pressure acoustics–frequency domain module coupled with solid mechanics and an acoustic-structure boundary was applied to the models. When acoustic waves propagate within the liquid medium, the longitudinal waves can be written as:


(2.1) $$\frac{1}{\rho_{0}c_{s}^{2}}\frac{\partial^{2}p}{\partial t^{2}}+\nabla ◼\left(-\frac{1}{\rho_{0}}\nabla p\right)=0,$$

where $c_{s}$ is the speed of sound (m s^−1^), p is the sound pressure (Pa). $\rho_{0}$ is the density (kg m^−3^), which is included in the equation because of its variations in different computational domains within the model. For the harmonic solution of the pressure $p\left(x,t\right)=p\left(x\right)e^{i\omega t}$ , with the angular frequency ω (rad s^−1^), equation (2.1) can be simplified as:


(2.2) $$\nabla ◼\left(-\frac{1}{\rho_{0}}\nabla p\right)-\frac{\omega^{2}p}{\rho_{0}c_{s}^{2}}=0.$$

While the acoustic waves interact with the solid medium, the multiphysics coupling provides and assigns the boundary conditions for the two-way acoustic structural coupling between the liquid (e.g. seawater and soft tissues) and the solid (e.g. skull and columella). The fluid–solid boundary condition includes the following interaction between fluid and solid domains:


(2.3) $$F=-n_{s}p,$$

(2.4) $$-n_{a}◼\left(-\frac{1}{\rho_{0}}\nabla p\right)=a_{n},$$

(2.5) $$a_{n}=\left(n_{a}◼u\right)\omega^{2},$$

where F is a pressure load (force per unit area) on the boundaries where the fluid interacts with the solid, $n_{s}$ is the outward-pointing unit normal vector seen from inside solid domain, $n_{a}$ is the outward-pointing unit normal vector seen from inside liquid, $a_{n}$ is normal acceleration of the solid surface in the liquid domain boundary and u is the calculated harmonic displacement vector of the solid structure.

### 2.4. Derivation of material properties

None of the material properties have been experimentally measured from Little Penguins but referring to existing literature and knowledge of anatomical similarities [36–38], physiologically relevant values for the properties can be estimated. For simplicity, all the bony structures (e.g. skull) and soft tissue components (e.g. muscles) were modelled as homogeneous, isotropic and linear. There were no measured values available for Young’s modulus and Poisson’s ratio for the bones and columella of the Little Penguin; therefore, the values commonly used for solid bone structures of vertebrates were used in this study [39]. The values of material properties set for the models are shown in table 1.

**Table 1.** The material properties for the medium and structures in the models.

material	sound speed (m s^−1^)	density (kg m^−3^)	Young’s modulus (MPa)	Poisson’s ratio
seawater	1483	998		
bony structures	3500	2.3 × 10^3^	60 × 10^3^	0.3
soft tissues	1500	1100		
inner ear	986	1466		
columella	3500	2.3 × 10^3^	35 × 10^3^	0.3

We performed sensitivity tests for the models by changing the material properties of the structures from −10% to +10%. Then we ran the simulation with the same settings and compared the results with the original data. For example, to determine the variation that could be attributed to the variable material properties, we calculated the correlation coefficient of several parameters across the frequencies (e.g. the columella footplate velocity and the sound pressure of the cochlea) between the two sets of data.

### 2.5. Transfer function and audiogram curve

The ear (i.e. external, middle and inner ear) can be seen as a series of components. Our FE models were accurately built based on the high-resolution microCT data, especially the ear canal, middle ear, columella and inner ear were constructed in detail. The synergistic response of these ear components to incoming acoustic signals together determines the audiogram [24,25,27]. When the incident acoustic wave impinges upon the animal’s head, it partially reflects off the skin and feathers and partially propagates further in the soft tissues (e.g. muscle). Some of the acoustic energy travels through the dense bony structures as elastic waves. The ears vibrate as a result of the incident acoustic wave $p_{input}$ , generating the motion of the columella within the oval window, which produces a velocity at the columella’s footplate ( $v_{cf}$ ). The motion of the columella’s footplate pushing in and out of the oval window drives the cochlea mechanically, causing the perilymph of the inner ear to vibrate. Based on this sound reception mechanism, the output of the model is a frequency-dependent transfer function between the magnitude of the velocity at the columella’s footplate ( $v_{cf}$ ) and amplitude of the input sound pressure ( $p_{input}$ ), giving a transfer function with the units of nm s^−1^ Pa^−1^.

The audiograms were predicted based on the transfer functions. It is a reliable method since both considers the motion of the solid materials and the sound pressure on the fluid materials in the animal’s head [27]. To calibrate the audiogram curve of the fin whale (*Balaenoptera physalus*), Cranford & Krysl [27] assumed its hearing threshold to be similar to that measured for bottlenose dolphins and killer whales (approx. 70 dB re. 1 μPa). Based on this, the minimum threshold pressure across all frequencies was estimated. Unfortunately, audiograms have not been measured for the Little Penguin (or any penguin species), neither IA nor UW; only the sensitivity range of Black-footed Penguins (only IA, 600–5000 Hz) was measured by Wever *et al*. [9] using the cochlear potential method. Therefore, this study referred to the values of the stapes velocity calculated at the threshold for other terrestrial species from the literature to convert the transfer function [24] and produce the final Little Penguin audiogram.

## 3. Results

### 3.1. Finite-element modelled sound pressure fields

**Figure 4.**
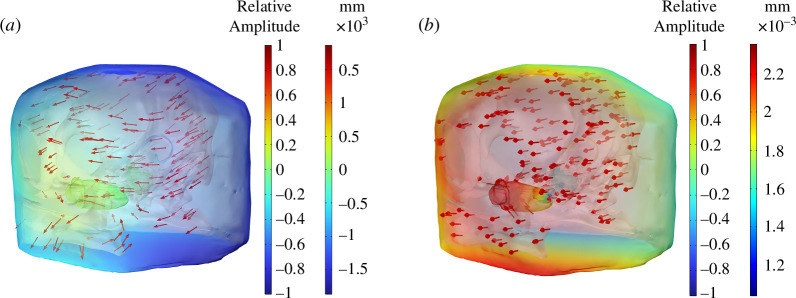
Sound pressure field of soft tissue and displacement field of the skull and columella in the (*a*) IA and (*b*) UW models (freq. = 1500 Hz). The colour bars indicate the relative amplitude of sound pressure and the relative magnitude of the displacement, respectively. The arrows denote the direction of the displacement.

The outputs of the FE model were temporal animations of the propagating acoustic pressure and response of anatomical structures. The incident acoustic stimulus had been excited from the left side of the penguin’s head. The acoustic wave travelled through the medium (air/seawater) surrounding the head and then interacted with the complex head structures (i.e. soft tissues, ear apparatus, skull, etc.). The elastic waves generated motion within the stiff and dense bony structures (e.g. skull and columella) causing deformation. The sound pressure and displacement fields in the penguin’s head were calculated at each of the excitation frequencies for both IA and UW models. Figure 4 shows a snapshot of the exported results at 1500 Hz. The relative sound pressure at the left side of the head was significantly higher than at the right side in both models because the left ear was closer to the sound source. The direction of the displacement in both models was towards the left side of the head. This was owing to the left side source excitation generating a force acting on the particles to restore them to their original position.

**Figure 5.**
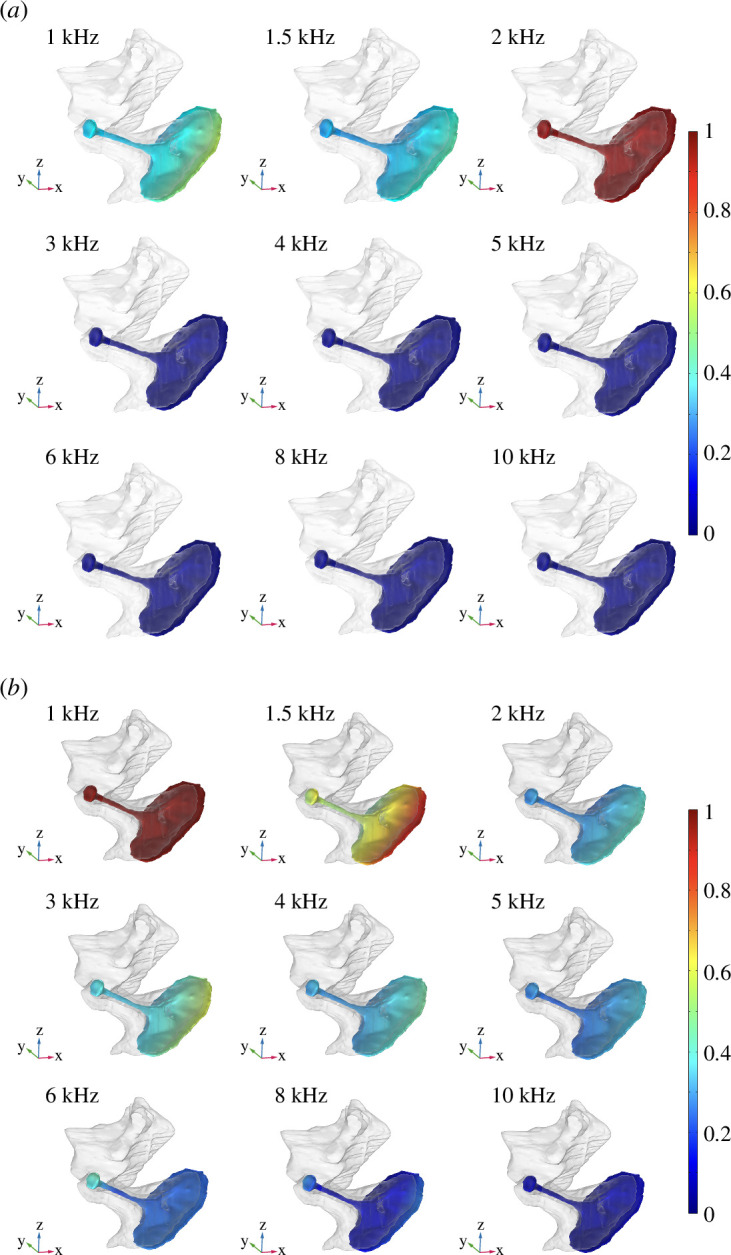
Motion of the columella for incident sounds from 1 to 10 kHz. The colour bar indicates the relative magnitude of the velocity. The transparent part represents the middle ear. (*a*) IA model. (*b*) UW model.

When the incident sound travelled through the ear canal, it caused the tympanic membrane to vibrate. The bony columella, located inside the middle ear, was set into motion by this vibration. The columella’s footplate sat in the oval window. The motion of the columella pushed the footplate in and out, driving the fluid of the cochlea and generating the vibration on the perilymph of the inner ear. To better examine the motion of the columella during sound reception, the columella in response to the incident acoustic waves in the frequency range from 1 to 10 kHz was compared in both models (figure 5). The results showed that the best frequency response range of the columella to the incoming sound was approximately 1−2 kHz for the IA model and 1−1.5 kHz for the UW model, respectively. In the IA model, with frequencies higher than 2 kHz, the motion of the columella was significantly reduced and became stationary. In the UW model, the motion was also reduced when the frequency was above 2 kHz. The columella became relatively stationary when the frequency was higher than 8 kHz.

### 3.2. Estimated transfer functions

**Figure 6.**
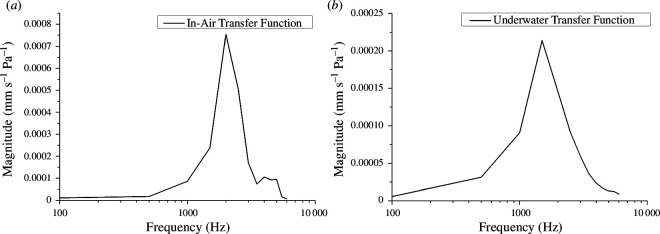
Transfer functions of the IA model (*a*) and the UW model (*b*).

The transfer functions between the amplitude of the incident sound pressure wave and the magnitude of the velocity of the columella’s footplate are shown in figure 6. The peak response frequencies of the IA and UW models were approximately 2000 and 1800 Hz, respectively. Both transfer functions attenuated sharply at higher frequencies. Early tape recordings showed that the ‘grunt’ and ‘bray’ sounds made by Black-footed Penguins had the most prominent frequencies below 2000 Hz (e.g. some of the ‘brays’ mainly contained frequencies between 1200 and 1700 Hz) [9]. Therefore, our results were consistent with vocalizations of the Black-footed Penguins.

### 3.3. Predicted in-air and underwater audiograms

**Figure 7.**
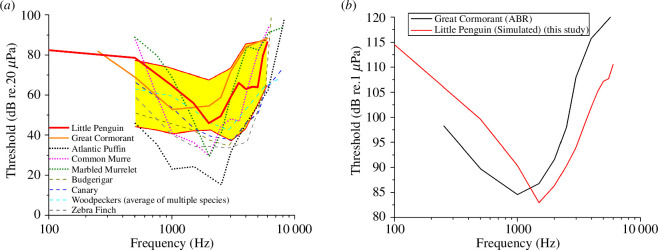
Predicted in-air (IA) and underwater (UW) audiograms of the Little Penguin. (*a*) Predicted IA audiogram, which is compared with the measured aerial hearing thresholds of other birds. The dotted lines denote the audiograms of several alcid diving birds: Atlantic Puffin *Fratercula arctica* [38], Common Murre *Uria aalge* [39] and Marbled Murrelet *Brachyramphus marmoratus* [40]. The dashed lines denote the audiograms of several terrestrial birds: Budgerigar *Melopsittacus undulatus* [41], canary *Serinus canaria* [42], woodpeckers (average of multiple species) [43] and Zebra Finch *Taeniopygia guttata* [44,45]. The solid lines denote non-alcid diving birds: Little Penguin (this study) and Great Cormorant *Phalacrocorax carbo sinensis* [14]. The yellow region shows the maximum/minimum auditory evoked potential (AEP) aerial hearing range of other measured non-alcid diving bird species from Crowell *et al*. [46]. The lower bound of the yellow region reflects the minimum thresholds across all those bird species, and the upper bound of the shaded region reflects the maximum thresholds. (*b*) Predicted UW audiogram of the Little Penguin, which is compared with the data measured for the Great Cormorant by Larsen *et al*. [14].

Based on the transfer functions, IA and UW audiograms were computed as shown in figure 7.

In air, the lowest predicted hearing threshold of the Little Penguin was approximately 46 dB re. 20 µPa at approximately 2 kHz with a gradual increase in thresholds at lower frequencies and a steeper increase at higher frequencies (>5 kHz). The region of best sensitivity (based on the lowest 30 dB range) was found to be approximately 550–5400 Hz. Wever *et al*. [9] used cochlear potential methods to study aerial hearing in Black-footed Penguins (also a small penguin species). Based on the results from three Black-footed Penguins, the best auditory acuity was found from 600 to 5000 Hz. Therefore, our predicted IA audiogram matched the data measured from the Black-footed Penguins. Furthermore, the shape of our IA audiogram curve was also similar to those of aerial audiograms of other bird species, including both diving and terrestrial birds (figure 7*a*), even though the lowest threshold of the Little Penguin was higher than those of the three alcid diving birds: Atlantic puffin [40], Common Murre [41] and Marbled Murrelet [42]. Penguins are spheniscids (non-alcid diving birds). Our predicted IA audiogram curve was closer to those of non-alcid diving birds. Our audiogram curve fell well into the maximum/minimum auditory evoked potential (AEP) audiograms of several non-alcid diving bird species from the previous study [47] and was comparable with the aerial ABR audiogram of the Great Cormorant [14].

Under water, the lowest predicted threshold was approximately 83 dB re. 1 µPa at approximately 1.5 kHz. The region of best sensitivity was approximately 200–6000 Hz. Above this range, the hearing sensitivity fell off precipitously. Unfortunately, very little is known about UW audiograms of diving birds; only one study by Larsen *et al*. [14] has measured the UW ABR audiogram for the Great Cormorant (the black curve in figure 7*b*). Our predicted UW audiogram showed a similar frequency-dependent pattern of sensitivity to the data from Larsen *et al*. [14], with a slightly lower sensitivity (approx. 2 dB) and a slightly higher sensitive frequency range (approx. 500 Hz).

In general, our predicted audiograms showed good agreement with those of other diving birds in both form and range of auditory sensitivity.

## 4. Discussion

### 4.1. Predicted audiograms

Larsen *et al*. [14] was, to our knowledge, the only study that provided a robust comparison of IA and UW hearing thresholds in a diving bird (Great Cormorant). Their results indicated that mean hearing thresholds across individuals were similar between the two environments, suggesting that the diving birds might share a similar trend in terms of IA and UW audiograms [41,42]. In our study, the two predicted audiograms of Little Penguins also showed a similar trend, supporting the assumption by Larsen *et al*. [14].

Our predicted IA audiogram is relatively less sensitive compared with those of alcid diving birds and terrestrial birds. We used the stapes velocity calculated at the threshold for other terrestrial species from the literature for the interpretation of the transfer function [24]. We ran some tests by using different values of stapes velocity from multiple terrestrial species to convert the transfer functions to audiograms, and the outcomes shifted approximately ±5 dB across the frequencies, which all fell well within the maximum/minimum hearing range of non-alcid diving birds in air. Moreover, it should be noted that the slope of our IA audiogram at the lower frequencies (below 500 Hz) was slightly different from the measured ones. This may be owing to the difference in species (e.g. anatomical difference) rather than a result of the missing beak in the FE models.

Sørensen *et al*. [8] documented detection of UW sound for Gentoo penguins. Their results found that Gentoo penguins had strong responses at 120 dB re. 1 µPa when an acoustic stimulus with frequencies between 200 and 6000 Hz was played in a large water tank. Early studies found that humans usually have strong behavioural responses when they hear sounds 50 dB above the hearing threshold. For marine mammals, strong behavioural reactions were observed when the received levels were greater than 100 dB above the hearing threshold [48]. Therefore, our predicted UW audiogram might be able to explain why there was no reaction recorded at 100 dB re. 1 µPa in the experiment by Sørensen *et al*. [8]: our predicted audiogram showed the lowest threshold was approximately 83 dB re. 1 µPa, and thus the 100 dB re. 1 µPa sounds might not be loud enough to elicit a reaction.

Early work found that odontocetes can actively change their hearing sensitivities when a warning sound precedes a loud sound [49]. To protect hearing, bats can also alter their hearing sensitivity during echolocation by contracting the stapedial muscle [50]. Our model did not take such active sensitivity changes into account because the exact mechanisms involved have yet to be determined [49]. Therefore, this study only predicted the anatomical hearing capabilities. It should be noted that additional muscular or neurological amplification or dampening [49] might shift (parts of) the audiograms in certain situations for Little Penguins.

### 4.2. The limitations of the models and future work

Note that the audiograms we predicted in this study are only approximations, there were some limitations that need to be addressed and improved in future studies. Owing to Little Penguins being very small (the length of an adult Little Penguin is only approx. 30 cm), it is difficult to collect large enough soft tissues for tissue property measurement. For example, measuring sound speed in the muscles using an ultrasonic velocimeter requires collecting multiple samples with a thickness of at least 1 cm, which is impossible for Little Penguins. There are also no relevant data available for any penguin species that could be used as a proxy. Therefore, we had to input values for tissue properties based on existing literature and knowledge of anatomical similarities, from other bird species [36–38]. The same methods were commonly used in previous FEA studies [24–28]. Based on the results of model sensitivity tests, we only found slight changes from the model outputs when the material properties varied. For instance, when the material properties of all structures were reduced by 10%, the correlation coefficients of the columella footplate velocity and the cochlea sound pressure between the two sets of outputs were 0.99 and 0.89, respectively; when the material properties of all structures were increased by 10%, the correlation coefficients of the columella footplate velocity and cochlea sound pressure between the two sets of outputs were 0.99 and 0.99, respectively. The results suggested very little sensitivity of our models.

Previous studies using FEA to estimate hearing thresholds of baleen whales have shown that different locations of source excitation could cause reasonably different response curves [24,25]. Therefore, it would be important to test this theory on the Little Penguin models in future. This requires to first perform microCT scans on a full penguin head. In this study, the beak was removed to fulfil the size requirement for microCT scanning. To avoid the effect of the missing beak, we only located the source excitation in front of the left ear. Early study [51] showed that birds without external ears tended to have better directional hearing on both sides of the head. Thus, locating the source excitation on the left side of the head in this study might be an ideal setting to predict hearing sensitivity and to compare with the measured data from other birds. Nevertheless, performing a full-head microCT scan for building a full-head sound reception FE model will be important future work.

### 4.3. Potential effects of anthropogenic noise on Little Penguins

The soundscape of many coastal areas is changing with humans increasingly using these areas, resulting in seabirds, including little penguins facing progressive acoustic habitat degradation. The modelling results here indicate that hearing frequencies of Little Penguins overlap with many anthropogenic noise sources, both in air and under water. For example, the noise sources in air, such as traffic noise, human speech, offshore constructions and passing aircraft are going to be audible to Little Penguins (see [52] for an overview of IA noise spectra). Under water, ship traffic is a primary source of noise between 20 and 1000 Hz [53]. In coastal areas near cities, boat noise is common [54]. Pile driving is common in coastal regions undergoing development, emitting broadband acoustic energy between 50 Hz and 5 kHz [55]. Another common noise source in the ocean are airgun signals from seismic surveys, with most acoustic energy below 500 Hz [56]. Military sonar typically ranges between 1 and 10 kHz [57]. It appears that these noises are detectable by Little Penguins, which may induce various behaviours. Our results indicate that this species is susceptible to disturbance from a range of anthropogenic noise types both in air and under water.

## 5. Conclusions

This was, to our knowledge, the first study that uses imaging-based FE modelling to predict auditory sensitivity in the Little Penguin both in air and under water. It is the only currently available method capable of predicting hearing sensitivities for sound reception in Little Penguins over a broad frequency range, between 100 Hz and 10 kHz. The results provide a valuable indication of the ranges of frequencies and thresholds that can be heard by Little Penguins. The predicted audiograms show good agreement with those of other diving birds in both form and range of auditory sensitivity. The study demonstrates important tools to study the sound reception process and predict auditory sensitivity for inaccessible animals, if an animal species shares a similar sound reception mechanism. Finally, this study provides critical information for marine conservation efforts as the outputs from this study can inform noise impact mitigation and conservation management strategies.

## Data Availability

The data were shared here: [58]. Supplementary material is available online [59].
